# Congestive Heart Failure 30-Day Readmission: Descriptive Study of Demographics, Co-morbidities, Heart Failure Knowledge, and Self-Care

**DOI:** 10.7759/cureus.18661

**Published:** 2021-10-11

**Authors:** Luai Madanat, Monique Saleh, Marina Maraskine, Alexandra Halalau, Florian Bukovec

**Affiliations:** 1 Internal Medicine, Beaumont Hospital, Royal Oak, USA; 2 Cardiovascular Medicine, Beaumont Hospital, Royal Oak, USA

**Keywords:** heart failure knowledge, heart failure readmissions, self-care behaviors, 30-day readmission, congestive heart failure

## Abstract

Background

Congestive heart failure (CHF) readmissions are associated with substantial financial and medical implications. We performed a descriptive study to determine demographic, clinical, and behavioral factors associated with 30-day readmission.

Materials and methods

Patients hospitalized with CHF at William Beaumont Hospital in Royal Oak, MI, from March 2019-May 2019 were studied. Response to heart failure knowledge and self-care questionnaires along with the patients’ demographic and clinical factors were collected. Thirty-day readmission to any of the eight hospitals in the Beaumont Health System was documented.

Results

One-hundred ninety-six (196) patients were included. The all-cause 30-day readmission rate was 23%. A numerical higher rate of readmissions was observed among males (23.7% vs 22.2%), current smokers (27.3% vs 22.9%), and patients with peripheral vascular disease (PVD; 28.9% vs 21.2%), diabetes mellitus (DM; 26.4% vs 18.9%), hypertension (HTN; 26.4% vs 10%), coronary artery disease (CAD; 24.6% vs 19%), and prior history of cerebrovascular accident (CVA; 28.9% vs 21.2%) (p>0.05). Reduced left ventricular ejection fraction (LVEF) was associated with higher readmissions (24.4% vs 20.5%, p=0.801). Patients with the highest reported questionnaire scores corresponding to better heart failure knowledge and self-care behaviors at home were readmitted at a similar rate compared to those scoring in the lowest interval (25%, p=0.681).

Conclusion

Though statistically insignificant due to the limitations of sample size, a higher percentage of readmissions was observed in male patients, current smokers, reduced LVEF, and higher comorbidity burden. Better reported patient self-care behavior, medication compliance, and heart failure knowledge did not correlate with reduced readmission rates. While the impact of medical comorbidities on 30-day readmissions is better established, the role of socioeconomic factors remains unclear and might suggest a focus for future work.

## Introduction

Background 

Per the American Heart Association, congestive heart failure (CHF) accounted for more than $30 billion of the US health care expenditure in 2012, with an estimated increase of 127% to $68.9 billion by 2030 [1]. CHF currently affects 6.2 million adults over the age of 20 in the United States with projections estimating that more than 8 million adults will have heart failure by 2030 [2]. Hospitalization rates in patients with CHF are estimated to be 18 per 100,000, leading to 700,000 inpatient admissions per year [3-5]. In addition to the economic implications, CHF remains one of the leading causes of morbidity and mortality. In 2017, the all-cause mortality in patients with CHF was reported to be 89.7 per 100,000, leading to 80,480 deaths in that year alone [6].

CHF is the most common cause of hospital readmissions among elderly and Medicare patients [2,7]. CHF readmissions receive particular interest given their pivotal effect on the total cost of heart failure. They are perceived as a correctable source of an unnecessary addition to morbidity, mortality, and health care expenditure. This has directed the Center for Medicare and Medicaid Services (CMS) and the Patient Protection and Affordable Act to mandate reporting of all-cause 30-day hospital readmissions as well as institute financial penalties for hospitals with the highest 30-day readmission rates respectively [8-9]. As defined by CMS, readmission is considered as any admission within 30 days of discharge from the same or another hospital [10]. According to Medicare, the median risk-standardized 30-day readmission rate for CHF patients hospitalized from 2009 to 2012 was 23% [2]. Other studies have reported readmission rates up to 50% within a one-year period [11-13]. Many of these readmissions are deemed preventable with literature estimating that 23.1% of hospital 30-day readmissions can be avoided [14-15]. 

Many risk factors have been identified as leading causes of hospital readmission in patients with CHF. In 2017, a study involving 155,146 patients utilized a statewide discharge data set from Pennsylvania to address different socioeconomic and medical factors contributing to increased risk of readmission from CHF. Male sex, age <65 years, African American descent, prolonged hospital stay, and several comorbidities, including a history of MI, COPD, renal failure, and DM, were associated with a higher risk for readmissions [16]. In a subsequent cohort study, including more than 1 million patients, the overall readmission rate was highest among individuals aged <65 years of age, African Americans, and those with a history of drug abuse and renal failure [17].

Tremendous efforts have been employed to reduce the rate of hospitalizations and readmissions, including remote monitoring, early drug initiation, and close follow-up [18]. A better understanding of potentially modifiable risk factors is needed if hospitals are to reduce CHF readmissions.

Objectives 

Our primary objective was to determine the 30-day readmission rates for patients initially admitted to the hospital with a principal diagnosis of CHF exacerbation and to highlight the demographic and clinical factors that predict a hospital 30-day readmission. We also aimed at determining the relationship between better heart failure knowledge and home self-care behaviors toward 30-day CHF readmission rates.

## Materials and methods

Study design

We conducted a retrospective cohort study on patients admitted to the hospital with a primary diagnosis of CHF exacerbation. The enrollment of subjects was based on the heart failure nurse-clinician population who see patients admitted to the hospital with a primary diagnosis of heart failure exacerbation. In addition, heart failure clinicians interview hospitalized patients and obtained a questionnaire centering on self-care and heart failure knowledge assessments to better assess social and medical aspects of their illness (Table 1). They then teach patients about heart failure and offer follow-up visits/phone calls at the time of discharge. A score interval was generated based on these questionnaire items. Each individual answer was given a score from 0 to 4. The total score range was between 0 and 28. Higher scores were presumed to insinuate better self-care management of their heart failure.

**Table 1:** Self-care and heart failure knowledge questionnaire CHF: congestive heart failure

Questionnaire Item Score	4	3	2	1	0
Blood pressure check at home	Everyday	Weekly	Monthly	Annually	Never
Weight check at home	Everyday	Weekly	Monthly	Annually	Never
Low salt diet	Everyday	Most days	Once a week	Once a month	Never
Miss medications	Never	Once a month	Once a week	Most days	Everyday
Visited the outpatient physician who manages my CHF in the last	Week	Month	Year	5 Years	Never
Interest in learning about CHF	Very interested	Interested	Somewhat interested	Little Interest	No interest
Knowledge about CHF	Excellent	Good	Average	Poor	Very poor

Setting

The study was done at Beaumont Hospital Royal Oak between March 2019 and May 2019. As part of a large health system, Beaumont Health has eight hospitals in the greater Detroit area, and hospital readmission within 30 days to any of the locations was included. Approval to conduct this study was obtained from the Institutional Review Board (IRB) at Beaumont Health System (IRB #2019-211).

Participants

We retrospectively identified patients who were admitted to the hospital with a primary diagnosis of CHF exacerbation. Inclusion criteria included: adults older than 18 years of age with a prior diagnosis of congestive heart failure. Exclusion criteria included patients with cognitive impairments leading to an inability to answer questions/participate in care, patients coming from a place other than home (rehab, extended care facility patients), and those with end-stage renal disease on dialysis.

Study size

All cases admitted to the hospital during the study period determined the sample size. A total of 196 patients met the inclusion criteria and were included in the analysis

Variables and data collection

Responses to the questionnaires, as well as demographic information (age, sex, race), comorbid conditions (diabetes, renal disease, hypertension, coronary artery disease, stroke, vascular disease, atrial fibrillation), number of medications prescribed at discharge, tobacco use history, and ejection fraction estimated from a recent echocardiogram, were collected by the research investigators and stored in RedCap, a secured web application used to form databases (Vanderbilt University, Nashville, Tennessee). A review of the electronic medical record 30 days post-discharge was carried out to determine if any-cause readmission had taken place. Readmissions were documented in RedCap along with the principal diagnosis of that admission.

Statistical methods

Readmission rates for demographics, medical comorbidities, heart failure knowledge, and self-care tasks were reported as percentages. We tested for an independent association of readmission rates using a chi-square test of independence. Fisher’s exact test was used where cell frequencies are <10. Unadjusted odds ratio (OR) was also used to demonstrate associations between demographic and clinical variables with 30-day readmission.

## Results

Patient characteristics

A total of 196 patients were involved in this study, distributed equally between both sexes (females 50.5%). The majority of patients were white (61.2%), with the remaining being African Americans (AA) (35.7%) or other ethnicities (3.1%). Most patients were 71-80 years of age (29.1%). The most common comorbidities observed among participants were hypertension (94.9%) and coronary artery disease (70.4%). Of the 196 patients, 22 (11.2%) were current tobacco smokers. Patients were classified according to left ventricular ejection fraction (LVEF) and subdivided into three groups: reduced LVEF <40%, moderately reduced LVEF 40-49%, and preserved LVEF defined as EF equal or greater than 50%. Eighty-six (86) patients (43.9%) had reduced EF, and 32 (16.3%) had moderately reduced EF (Table 2).

**Table 2:** Patient demographic and clinical characteristics

Patient Characteristic	Frequency
Age	
31-40	4 (2%)
41-50	13 (6.6%)
51-60	22 (11.2%)
61-70	36 (18.4%)
71-80	57 (29.1%)
81-90	50 (25.5%)
91-100	14 (7.1%)
Sex	
Female	99 (50.5%)
Male	97 (49.5%)
Race	
Black	70 (35.7%)
White	120 (61.2%)
Other	6 (3.1%)
Tobacco use	
Current user	22 (11.2%)
Past user	91 (46.4%)
Never used	83 (42.3%)
Medical conditions	
Diabetes	106 (54.1%)
Chronic kidney disease	111 (56.6%)
Hypertension	186 (94.9%)
Coronary artery disease	138 (70.4%)
Stroke	51 (26.0%)
Vascular disease	45 (23.0%)
Atrial fibrillation	111 (56.6%)
Number of medications at discharge	
1 - 5	10 (5.1%)
6-10	67 (34.2%)
11-15	70 (35.7%)
16-20	34 (17.3%)
21-25	11 (5.6%)
26-30	4 (2.0%)
Ejection fraction	
10 - 19%	9 (4.6%)
20 - 29%	39 (19.9%)
30 - 39%	38 (19.4%)
40 - 49%	32 (16.3%)
50 - 59%	26 (13.3%)
60 - 69%	43 (21.9%)
70% +	9 (4.6%)
Total number of patients	196

Outcome data

In total, 23.0% (45 patients) had any-cause readmission regardless of age, sex, social, or comorbid medical conditions. Of the 23% who were readmitted, 62% were due to CHF exacerbation (Table 3).

**Table 3:** Thirty-day readmission rates based on patient demographic and clinical factors P-values for chi-square test of independence

Total number of patients 196	Total readmitted 45 (23%)	P-value
Sex		0.804
Female	22 (22.2%)	
Male	23 (23.7%)	
Age		0.719
31-40	1 (25.0%)	
41-50	1 (7.7%)	
51-60	5 (22.7%)	
61-70	9 (25.0%)	
71-80	11 (19.3%)	
81-90	15 (30.0%)	
91-100	3 (21.4%)	
Race		0.667
Black	14 (20.0%)	
White	29 (24.2%)	
Other	2 (33.3%)	
Tobacco use		0.867
Current user	6 (27.3%)	
Past user	20 (22.0%)	
Never used	19 (22.9%)	
History of medical conditions		
Diabetes		0.212
Yes	28 (26.4%)	
No	17 (18.9%)	
Chronic kidney disease		0.868
Yes	25 (22.5%)	
No	20 (23.5%)	
Hypertension		0.317
Yes	44 (23.7%)	
No	1 (10.0%)	
Coronary artery disease		0.389
Yes	34 (24.6%)	
No	11 (19.0%)	
Stroke		0.910
Yes	12 (23.5%)	
No	33 (22.8%)	
Vascular disease		0.281
Yes	13 (28.9%)	
No	32 (21.2%)	
Atrial fibrillation		0.611
Yes	24 (21.6%)	
No	21 (24.7%)	
Number of medications at discharge		0.755
1 - 5	2 (20.0%)	
6-10	15 (22.4%)	
11-15	13 (18.6%)	
16-20	11 (32.4%)	
21-25	3 (27.3%)	
26-30	1 (25.0%)	
Ejection fraction		0.801
Reduced: < 40%	21 (24.4%)	
Mod Red: 40 - 49%	8 (25.0%)	
Preserved: > 49%	16 (20.5%)	

Readmission rates based on demographic and social factors, medical comorbidities, and ejection fraction

Males had a readmission rate of 23.7%, compared to females 22.2% (p=0.804). African Americans had a readmission rate of 20%, Caucasians 24.2%, with individuals of other ethnicities readmitted at a rate of 33.3% (p=0.667). The highest rate of readmission was observed among the age group 81-90 years (30%). However, increasing age did not correlate with higher readmission rates (p=0.719). The rate of 30-day readmission among current smokers (27.3%) was higher than that among non-smokers (22.9%) and individuals with a previous history of tobacco use (22%, p=0.869).

A higher percentage of patients with diabetes (26.4% vs 18.9%), hypertension (23.7% vs 10%), coronary artery disease (24.6% vs 19%), stroke (23.5% vs 22.8%), and vascular disease (28.9% vs 21.2%) compared to those who did not have these diagnoses (p>0.05). The rate of readmission was not higher in patients with chronic kidney disease (22.5%) and atrial fibrillation (21.6%) when compared to patients without (23.5% and 24.7%, respectively, P>0.05). The readmission rates were highest among patients with moderately reduced LVEF (25%), followed by reduced LVEF (24.4%) and preserved LVEF (20.5%) (p=0.801). A higher number of prescribed medications at the time of the discharge did not correlate with higher readmission rates (p=0.755) (Table 3).

Unadjusted odds ratio (OR) of readmission was calculated for demographic and clinical variables with higher readmission rates. Males (OR 1.08, p=0.804), current smokers (OR 1.26, p=0.668), and patients with diabetes (OR 1.54, p=0.214), hypertension (OR 2.79, p=0.337), coronary artery disease (OR 1.40, p=0.390), peripheral arterial disease (PAD) (1.51, p=0.283), and stroke (OR 1.04, p=0.910) were more likely to be readmitted. Patients with reduced LVEF (OR 1.25, p=0.597) and moderately reduced LVEF (OR 1.29, p=0.605) were more likely to be readmitted compared to patients with preserved ejection fraction. Compared to individuals of white ethnicity, African Americans (OR 0.78, p=0.661) were less likely to be readmitted while those of other ethnicities (OR 1.57, p=0.614) had the highest odds of readmissions. Atrial fibrillation (OR 0.80, p=0.611) and CKD (OR 0.94, p=0.868) were not associated with higher readmissions.

Readmission rates based on self-care and heart failure knowledge questionnaire

Patients who followed a low salt diet daily had lower readmission rates compared to those who do not (21.4% vs 25%, p = 0.919). Daily weight measurements at home did not improve readmission rates (p=0.002). Patients’ self-reported knowledge about heart failure knowledge and interest in learning more about their condition did not prove to have lower readmission rates. Those who are compliant with their medications did not have fewer readmission rates (Table 4).

**Table 4:** Readmission rate based on self-care and heart failure knowledge questionnaire P-value for chi-square test of independence. Fisher’s exact test was used where cell frequencies are <10.

Questionnaire Item	Frequency (%)	Readmission rate (%)	P-value
Blood pressure check at home			0.002
Everyday	49 (25.0)	30.6	
Weekly	40 (20.4)	2.5	
Monthly or never	107 (54.6)	27.1	
Weight check at home			0.449
Everyday	76(38.8)	27.6	
Weekly	57 (29.1)	21.1	
Monthly or Never	63 (32.1)	19.0	
Low salt diet			0.919
Everyday	56 (28.6)	21.4	
Most days	100 (51)	23.0	
Once a week or Never	40 (20.4)	25.0	
Miss medication			0.234
Everyday	9 (4.6)	0.0	
Most days	12 (6.1)	8.3	
Once a week	28 (14.3)	25.0	
Once a month or Never	147 (75.0)	25.2	
Visited an outpatient physician who manages my CHF in the last			0.158
Week	1 (0.5)	100	
Month	137 (69.9)	24.8	
Year	52 (26.5)	19.2	
5 years or Never	6 (3.1)	0.0	
Interest in learning about CHF			0.514
Very interested	55 (28.1)	21.8	
Interested	62 (31.6)	25.8	
Somewhat interested	54 (27.6)	25.9	
No interest	25 (12.8)	12.0	
Knowledge about CHF			0.672
Excellent	21 (10.7)	28.6	
Good	80 (40.8)	20.0	
Average	68 (34.7)	26.5	
Poor or very poor	27 (13.8)	18.5	
Questionnaire score interval			0.681
0 – 7	4 (2.0)	25	
8 – 14	28 (14.3)	14.3	
15 – 21	116 (59.2)	24.1	
22 – 28	48 (24.5)	24.5	

Patients’ readmissions were also analyzed based on the score interval generated from the questionnaire responses (total score range 0-28). Higher scores insinuated better heart failure knowledge and self-care practice. The mean score was 18.4, median 19 (SD 4.3). A score interval of 15-21 range was the highest percentage achieved based on the patient’s questionnaire responses at 59.2%, followed by a score interval of 22-28, achieved by 24.5% of patients. A score interval of 8-14 and 0-7 was achieved by 14.3% and 2.0% of patients, respectively. Higher score intervals did not correlate with lower readmission rates. Patients with the highest score interval of 22-28 were readmitted at similar rates compared to patients scoring at the lowest interval 0-7 (25%, p=0.68) (Table 4).

## Discussion

In our study, the 30-day all-cause readmission rate was 23%, concurrent with other reports on CHF readmission rates among Medicare beneficiaries [2]. Males and individuals of a race other than Caucasian or African American were readmitted at higher rates than their counterparts. This is similar to results reported by Chamberlain et al., in which male gender (OR 1.11; 95% CI, 1.09-1.14) and race other than African American or Caucasian (OR 1.34; 95% CI, 1.29-1.38) were independent risk factors for hospital readmission [17]. Higher readmission rates were observed among co-morbidities, including diabetes, hypertension, coronary artery disease, vascular disease, and history of stroke. These findings are consistent with previous reports demonstrating higher readmissions with greater comorbidity burden [16-19]. Several other studies have provided a consistent association between a history of stroke and diabetes with re-hospitalization in patients with CHF [20-22]. Tobacco use has been previously linked to multiple readmission [23], and in our study, smokers had a higher readmission rate compared to non-smokers. Patients with reduced ejection fraction were readmitted at higher rates when compared to those with preserved EF, similar to previously published reports [24-25]. However, more recent publications demonstrate similar risks between both groups [26-27].

One important topic of interest was readmission rates based on the patient’s responses to the self-care and heart failure knowledge questionnaire. Patient self-management is believed to be a cornerstone of CHF outcomes [28]. Poor patient adherence to adequate self-care behavior has been associated with hospital readmissions [29-30]. Health literacy has also been linked to increased hospital admissions and mortality in patients with CHF [31-32], which could be in part due to poor patient ability to self-manage [33]. Several studies have demonstrated the effectiveness of heart failure self-care interventions in improving patient management behaviors and clinical outcomes [34-37]. In this study, daily adherence to blood pressure monitoring, weight check, and low salt intake was observed in 25%, 38.8%, and 25% of patients, respectively. More than half of the patients reported poor compliance with measuring blood pressure at home despite the education and emphasis placed on blood pressure readings at home by our heart failure nurse clinicians at discharge. Failure to adhere and comply with treatment plans is quite common in patients with CHF with the literature reporting non-adherence rates as high as 60% and 80% for medication regimens, and recommended lifestyle changes, respectively [28-30,33]. Adequate adherence to medications was reported in 75% of patients, similar to previous reports [38-39]. Better reported self-care practices, including daily blood pressure measurements, weight checks, and adherence to medications were not associated with lower readmission rates. Self-reported CHF knowledge and interest in learning about their illness did not correlate with lower readmission rates. Our results are consistent with several randomized controlled trials conducted to assess outcomes following patient self-management interventions. Although monitoring symptoms and compliance may have improved, there was no benefit in terms of admission and overall mortality [40-44].

More than half of our patients rated their knowledge about heart failure as excellent or good. However, this may not accurately represent a patient's true understanding of their illness. It is possible that a patient's subjectivity could have affected their responses and that these results do not accurately reflect patient management, adherence to treatment and recommendations, or knowledge of their disease process. Despite this additional bedside teaching, the readmission rates were similar to previously reported studies. It is worth mentioning that socioeconomic factors, such as marital status, occupation, and income, also play a vital role in the rates of readmission, as demonstrated in previous studies [45-46]. These factors were not investigated in this study and could have influenced the outcome of readmission rates.

Attempting to identify factors that predict hospital readmission is pertinent in decreasing readmission rates and similarly for improving patient outcomes and decreasing the number of healthcare costs. Many risk factors have been previously identified as putting patients at higher risk for readmission, and various interventions have been implemented as an attempt to reduce hospitalizations.

Limitations

The current study has several limitations. It is a single-center retrospective study with a small sample size, and thus, statistical significance was difficult to achieve. Secondly, a patient's self-reporting was the primary source of data for the questionnaire, thus recall bias and a patient’s concern to reveal accurate information may have affected the results of this study. Chart review as the primary way to collect data also may have missed readmissions to hospitals outside the Beaumont Health System. The majority of patients were of white ethnicity; hence, this brings forth the issue of generalizability. In contrast to the general CHF population, these patients were all provided with extra CHF teaching as well as offered close follow-up via telephone or in-person visits with the CHF nurse-clinician clinic.

## Conclusions

This study was limited by sample size and thus statistical significance was not achieved. A higher numerical readmission rate was observed among males, current smokers, and in patients with hypertension, CAD, diabetes, vascular disease, a history of stroke, and chronic kidney disease. Improved patient self-care behaviors and heart failure knowledge did not correlate with reduced readmission rates. Although many of our results concerning comorbid conditions seem to align with previous study findings, it is necessary to perform further analyses of components that affect patients' admissions due to CHF, particularly those aspects that pertain to their care, adherence to treatment plans, and disease knowledgeability at home. The role of socioeconomic factors in rates of readmission is often overlooked and the future focus needs to be directed towards a more comprehensive patient approach. To our knowledge, there is no single effective prediction model that is used to help stratify patients based on the risk of readmission. Patient management and follow-up should be individualized based on social, demographic, and medical aspects.
